# Effect of Food Regulation on the Spanish Food Processing Industry: A Dynamic Productivity Analysis

**DOI:** 10.1371/journal.pone.0128217

**Published:** 2015-06-09

**Authors:** Magdalena Kapelko, Alfons Oude Lansink, Spiro E. Stefanou

**Affiliations:** 1 Institute of Applied Mathematics, Department of Logistics, Wroclaw University of Economics, Wroclaw, Poland; 2 Business Economics Group, Wageningen University, Wageningen, The Netherlands; 3 Food and Resource Economics Department, University of Florida, Gainesville, United States of America; 4 Business Economics Group, Wageningen University, Wageningen, The Netherlands; Agricultural University of Athens, GREECE

## Abstract

This article develops the decomposition of the dynamic Luenberger productivity growth indicator into dynamic technical change, dynamic technical inefficiency change and dynamic scale inefficiency change in the dynamic directional distance function context using Data Envelopment Analysis. These results are used to investigate for the Spanish food processing industry the extent to which dynamic productivity growth and its components are affected by the introduction of the General Food Law in 2002 (Regulation (EC) No 178/2002). The empirical application uses panel data of Spanish meat, dairy, and oils and fats industries over the period 1996-2011. The results suggest that in the oils and fats industry the impact of food regulation on dynamic productivity growth is negative initially and then positive over the long run. In contrast, the opposite pattern is observed for the meat and dairy processing industries. The results further imply that firms in the meat processing and oils and fats industries face similar impacts of food safety regulation on dynamic technical change, dynamic inefficiency change and dynamic scale inefficiency change.

## Introduction

In the past decade a series of food crises such as Bovine spongiform encephalopathy, Dioxin and foot-and-mouth disease challenged the suitability of current food industry safety schemes. In response, the European Union (EU) regulation coined the General Food Law (Regulation (EC) No 178/2002) was announced in January 2002, whose overall intent was to ensure food quality and safety for food stuff intended for human and animal consumption with the aims to protect a) consumers against fraudulent or deceptive commercial practices and b) the health and well-being of animals, plants and the environment [1]. The responsibility for implementing the General Food Law falls on the food operators and activities at the Member State level. Food operators bear responsibility for ensuring traceability of products at all stages of food production, processing and distribution and are required to remove harmful food stuffs immediately and inform authorities. Each Member State manages the liaison activities for the Rapid Alert System, Crisis Management Plan and liaison with the European Food Safety Authority.

With the regulation placing the main responsibility to food business operators in implementing food law requirements, changes in processes and/or equipment are needed to meet the regulatory requirements. These changes can necessitate increase in the cost of production and investment in new equipment. The economic performance of the European food manufacturers can be seriously altered in terms of the resulting productivity changes and extracting the maximum potential (i.e., the efficiency of production) of new processes and technologies now put into place.

Over the past decade, the performance of Spanish food manufacturing sector was challenged by the introduction of aforementioned EU regulation. Coping with this more stringent regulatory climate, Spanish food manufacturing firms had to undertake additional investments and deal with more administrative compliance [2], [3]. The implementation of this regulation is associated with increasing production costs, which can reduce the productivity of the food industry. With productivity growth often viewed as a longer term measure of economic performance, the substantial regulatory changes over the first decade of 2000 bring into question the appropriateness of using a long-run equilibrium framework to measuring performance.

The food industry is an important sector for the Spanish economy as it represents 16% of the net sales of industry, 17% of industrial employment and 8% of Spanish GDP in 2010. Its importance is further emphasized by the fact that it is one of the main exporting sectors of Spain. Meat processing is the main subsector within the food industry as measured by annual net turnover, followed by dairy products, and oils and fats products. The food industry in Spain is characterized by a predominance of small- and medium-sized enterprises [3], [4], which makes it vulnerable to external competition and potentially cost-increasing regulatory policies. This exposure to external competition is expected to increase following the ongoing globalization and the liberalization of food markets.

Research on the impact of regulation on productivity and productivity growth has a long tradition in the economic literature. Much of the existing research within this line analyses environmental regulation and focuses on examining the Porter hypothesis which suggests that environmental regulation may have a positive impact on firms’ performance as it can induce innovation [5], [6]. The earliest empirical attempt to analyse the effect of environmental regulations on productivity is [7] finding that abatement requirements impede the average capital and labour productivity in the U.S. paper, chemicals and primary metals industries. Subsequent studies at both the aggregate and industry levels find environmental regulations to be productivity reducing [8], productivity enhancing impacts of environmental regulation [9], [10], and even providing evidence of the positive impact of firms’ exposure to competition in inducing the productivity gains [11]. For the food manufacturing industry, [12] finds that the productivity of the Mexican food processing industry was increasing with the pressure of regulation, and the study of [13] confirms the productivity decline and technical regress in French food processing industry following this EU regulation. The literature on assessing the impact of regulation on productivity growth to date has focused on static measures of productivity. The shortcoming of the static approach is that it does not account for the disequilibrium of capital factors and may not appropriately reflect productivity growth and its components when substantial investments are undertaken.

The objective of this article is to assess the impact of the introduction of the General Food Law (Regulation (EC) No 178/2002) in 2002 on dynamic productivity growth and its components in the Spanish meat, dairy, and oils and fats industries. This article contributes to the literature by using a dynamic production framework to analyse the effects of regulation using the dynamic Luenberger productivity indicator [14]. The second contribution of this article is developing a decomposition of the dynamic Luenberger into the contributions of dynamic technical inefficiency change, dynamic scale inefficiency change and dynamic technical change in the nonparametric framework and assessing the impact of regulation to these components. When accounting for dynamic productivity growth at the firm level, the adaptation to the regulation can be elicited through the impact of various components of productivity growth. Regulation adopted today will affect firms’ productivity and its components into the future. This study measures the impact of regulation on dynamic productivity growth, dynamic technical change, dynamic technical inefficiency change and dynamic scale inefficiency change in each year following the introduction of the regulation. For this purpose, we apply the OLS bootstrap regression.

The next section develops the measures of dynamic productivity growth and its decomposition and then briefly presents the method to analyse the impact of regulation. This is followed by a description of the data of Spanish meat processing, dairy processing and oils and fats firms. The section to follow presents the results of dynamic productivity and its decomposition and the findings on the impact of regulation. The final section offers concluding comments.

## Methods

### The Luenberger Indicator of Dynamic Productivity Growth

The setting for a dynamic production decision making framework involves current decisions can impact future production possibilities. The dynamic framework of productivity growth is based on the production technology that relates at time *t* the vectors of variable inputs, **x**_*t*_, gross investments, **I**_*t*_ (which is the change in quasi fixed factors), and quasi-fixed factors, **k**_*t*_, to the production of vector of output, **y**_*t*_. In a dynamic approach the source of the intertemporal link of production decisions is adjustment costs connected with changes in the level of quasi-fixed factors. The adjustment costs can be defined as transaction or reorganization costs that may be either internally or externally driven [15], [16]. The theory of adjustment costs is developed in [17], [18], [19].

The production input requirement set is defined by [20] as *V*_*t*_(**y**_*t*_:**k**_*t*_) = {(**x**_*t*_,**I**_*t*_) can produce **y**_*t*_, given **k**_*t*_}, and it is assumed to have the following properties: *V*_*t*_(**y**_*t*_:**k**_*t*_) is a closed and nonempty set, has a lower bound, is positive monotonic in variable inputs **x**_*t*_, negative monotonic in gross investments **I**_*t*_, is a strictly convex set, output levels **y**_*t*_ increase with quasi-fixed inputs **k**_*t*_ and are freely disposable. [20] demonstrate these properties can support the technology be represented as a series of linearly inequality constraints. The property related to gross investments implies that there is a positive cost when investment in quasi-fixed inputs occurs; hence, it explicitly incorporates the adjustment costs.

The construction of economic performance measures in a dynamic context is built on the production technology that allows for adjustment costs. The Luenberger indicator of dynamic productivity growth is based on the dynamic directional distance function, which is an extension of the static directional distance function [21]. Directional distance function is a version of the Luenberger’s benefit function [22] from consumer theory that is applied in production theory. The input-oriented dynamic directional distance function with directional vectors for inputs (**g**_*x*_) and investments (**g**_*I*_), ${\vec{D}}_{t}^{i}(y_{t},k_{t},x_{t},I_{t};g_{x},g_{I})$, measuring dynamic technical inefficiency for each firm, is defined as follows:

$$\begin{array}{l} {\vec{D}}_{t}^{i}(y_{t},k_{t},x_{t},I_{t};g_{x},g_{I})=\max\left\{\beta \in ℜ:(x_{t}-\beta g_{x},I_{t}+\beta g_{I})\in V_{t}(y_{t}:k_{t})\right\},\\ \;g_{x}\in {ℜ}_{++}^{N},\;g_{I}\in {ℜ}_{++}^{F},(g_{x},g_{I})\neq (0^{N},0^{F}) \end{array}$$ (1)

if (**x**_*t*_ – *β* **g**_*x*_,**I**+*β* **g**_*I*_) ∈ *V*_*t*_(**y**_*t*_:**k**_*t*_) for some *β*, ${\vec{D}}_{t}^{i}(y_{t},k_{t},x_{t},I_{t};g_{x},g_{I})=-\infty$, otherwise. The subscript *i* refers to the index for inputs. This distance function measures the maximal translation of (**x**_*t*_,**I**_*t*_) in the direction defined by the vector (**g**_*x*_,**g**_*I*_), that keeps the translated input combination interior to the set *V*_*t*_(**y**_*t*_:**k**_*t*_). Because *β* **g**_*x*_ is subtracted from **x**_*t*_ and *β* **g**_*I*_ is added to **I**_*t*_, the dynamic directional distance function is defined by simultaneously contracting variable inputs and expanding gross investments. Silva and Oude Lansink (2013) proves that ${\vec{D}}_{t}^{i}(y_{t},k_{t},x_{t},I_{t};g_{x},g_{I})\geq 0$ fully characterizes the input requirement set, *V*_*t*_(**y**_*t*_:**k**_*t*_), therefore it is an alternative primal representation of the adjustment cost production technology. More details with regard to the dynamic directional distance function can be found in [23], [24], [25].

Extending the static Luenberger indicator of productivity growth defined by [21] to the dynamic setting assuming constant returns to scale (CRS) leads to the dynamic Luenberger productivity change indicator:

$$L(\cdot )=\frac{1}{2}\left\{\begin{array}{l} \left[{\vec{D}}_{t+1}^{i}(y_{t},k_{t},x_{t},I_{t};g_{x},g_{I})-{\vec{D}}_{t+1}^{i}(y_{t+1},k_{t+1},x_{t+1},I_{t+1};g_{x},g_{I})\right]\\ +[{\vec{D}}_{t}^{i}(y_{t},k_{t},x_{t},I_{t};g_{x},g_{I})-{\vec{D}}_{t}^{i}(y_{t+1},k_{t+1},x_{t+1},I_{t+1};g_{x},g_{I})] \end{array}\right\}$$ (2)

This indicator provides the arithmetic average of productivity change measured by the technology at time *t+*1 (i.e., the first two terms in (2)) and the productivity change measured by the technology at time *t* (i.e., the last two terms in (2)). The positive (negative) value of dynamic Luenberger indicates growth (decline) in productivity between *t* and *t+1*.

[14] use the dynamic directional distance function to decompose the Luenberger indicator of dynamic productivity growth into the contributions of dynamic technical inefficiency change (Δ*TEI*) and dynamic technical change (Δ*T*):

L(⋅)=ΔTEI+ΔT (3)

In this article we summarize the decomposition. The Appendix (S1 Appendix) provides more details on how to generate the Luenberger productivity measure and its decomposition.

Dynamic technical inefficiency change is defined as the difference between the value of the dynamic directional distance function at time *t* and time *t+*1:

$$\Delta TEI={\vec{D}}_{t}^{i}(y_{t},k_{t},x_{t},I_{t};g_{x},g_{I})-{\vec{D}}_{t+1}^{i}(y_{t+1},k_{t+1},x_{t+1},I_{t+1};g_{x},g_{I})$$ (4)

Dynamic technical change is computed as the arithmetic average of the difference between the technology (represented by the frontier) at time *t* and time *t+*1, evaluated using quantities at time *t* and time *t+*1:

$$\Delta T=\frac{1}{2}\left\{\begin{array}{l} \left[{\vec{D}}_{t+1}^{i}(y_{t},k_{t},x_{t},I_{t};g_{x},g_{I})-{\vec{D}}_{t}^{i}(y_{t},k_{t},x_{t},I_{t};g_{x},g_{I})\right]\\ +[{\vec{D}}_{t+1}^{i}(y_{t+1},k_{t+1},x_{t+1},I_{t+1};g_{x},g_{I})-{\vec{D}}_{t}^{i}(y_{t+1},k_{t+1},x_{t+1},I_{t+1};g_{x},g_{I})] \end{array}\right\}$$ (5)

Building on [14], the dynamic Luenberger measure can be further decomposed to allow for scale inefficiency change (Δ*SEI*) which requires relaxing the technology assumptions of constant returns to scale to permit variable returns to scale (VRS).

From a primal perspective, the dynamic technical inefficiency change component in Eq (3) can be decomposed as follows:

ΔTEI=ΔPEI+ΔSEI

$$\Delta PEI={\vec{D}}_{t}^{i}(y_{t},k_{t},x_{t},I_{t};g_{x},g_{I}|\;VRS)-{\vec{D}}_{t+1}^{i}(y_{t+1},k_{t+1},x_{t+1},I_{t+1};g_{x},g_{I}|\;VRS)$$

$$\begin{array}{l} \Delta SEI={\vec{D}}_{t}^{i}(y_{t},k_{t},x_{t},I_{t};g_{x},g_{I}|\;CRS)-{\vec{D}}_{t}^{i}(y_{t},k_{t},x_{t},I_{t};g_{x},g_{I}|\;VRS)\\ -\left[{\vec{D}}_{t+1}^{i}(y_{t+1},k_{t+1},x_{t+1},I_{t+1};g_{x},g_{I}|\;CRS)-{\vec{D}}_{t+1}^{i}(y_{t+1},k_{t+1},x_{t+1},I_{t+1};g_{x},g_{I}|\;VRS)\right] \end{array}$$ (6)

where *ΔPEI* is dynamic technical inefficiency change under variable returns to scale and *ΔSEI* is dynamic scale inefficiency change. Dynamic scale inefficiency change measures the difference between period *t* and period *t+1* regarding the comparison of the dynamic directional distance functions gauged relative to CRS technology with that relative to the VRS technology. Summarizing, the final decomposition of dynamic Luenberger indicator of productivity growth is obtained as follows:

L(⋅)=ΔT+ΔPEI+ΔSEI (7)

The positive (negative) values of components of dynamic Luenberger indicate the positive (negative) contributions of these components to dynamic productivity growth. For example, a positive value of dynamic technical inefficiency change implies a positive contribution of dynamic technical inefficiency change to dynamic productivity growth i.e., inefficiency decreased between *t* and *t+1*.

The empirical implementation of the dynamic directional distance functions which form the dynamic Luenberger indicator and its components is done using the nonparametric method of Data Envelopment Analysis (DEA) [26], [27]. Building on the results in [20], the following DEA model can be estimated to compute the dynamic directional distance function for time *t* in CRS technology:

$$\begin{array}{l} {\vec{D}}_{t}^{i}(y_{t},k_{t},x_{t},I_{t};g_{x},g_{I}|CRS)=\underset{\beta ,\gamma }{\max}\;\beta \\ \;s.t.\\ \;y_{t\;m}^{}\leq \sum_{j=1}^{J}\gamma^{j}y_{t\;m}^{j},\;m=1,\ldots ,M;\\ \;\sum_{j=1}^{J}\gamma^{j}x_{t\;n}^{j}\leq x_{t\;n}^{}-\beta g_{x_{n}},\;n=1,\ldots ,N;\\ \;I_{t\;f}^{}+\beta g_{I_{f}}-\delta_{f}k_{t\;f}^{}\leq \sum_{j=1}^{J}\gamma^{j}(I_{t\;f}^{j}-\delta_{f}k_{t\;f}^{j}),\;f=1,\ldots ,F;\\ \;\gamma^{j}\geq 0,\;j=1,\ldots ,J. \end{array}$$ (8)

where **γ** is an intensity vector, and *δ* is the rate of capital depreciation which is specific to each firm. The directional distance function in (8) is dynamic as it is a function of the change in the capital stock rather than the actual level of the capital stock.

Note that the dynamic directional distance function for time *t+1* ${\vec{D}}_{t+1}^{i}(y_{t+1},k_{t+1},x_{t+1},I_{t+1};g_{x},g_{I}|CRS)$ is obtained using the analogous linear program to (8). The mixed period dynamic directional distance function which projects the quantities at time *t+1* on the CRS technology at time *t* is given by:

$$\begin{array}{l} {\vec{D}}_{t}^{i}(y_{t+1},k_{t+1},x_{t+1},I_{t+1};g_{x},g_{I}|CRS)=\underset{\beta ,\gamma }{\max}\;\beta \\ \;s.t.\\ \;y_{t\;m}^{}\leq \sum_{j=1}^{J}\gamma^{j}y_{t+1\;m}^{j},\;m=1,\ldots ,M;\\ \;\sum_{j=1}^{J}\gamma^{j}x_{t+1\;n}^{j}\leq x_{t\;n}^{}-\beta g_{x_{n}},\;n=1,\ldots ,N;\\ \;I_{t\;f}^{}+\beta g_{I_{f}}-\delta_{f}k_{t\;f}^{}\leq \sum_{j=1}^{J}\gamma^{j}(I_{t+1\;f}^{j}-\delta_{f}k_{t+1\;f}^{j}),\;f=1,\ldots ,F;\\ \;\gamma^{j}\geq 0,\;j=1,\ldots ,J. \end{array}$$ (9)

The mixed period dynamic distance function ${\vec{D}}_{t+1}^{i}(y_{t},k_{t},x_{t},I_{t};g_{x},g_{I}|CRS)$, which projects quantities in period *t* on the CRS technology in period *t+1*, is obtained analogously to (9). Finally, note that to estimate the dynamic directional distance functions for VRS technology, the constraint $\sum_{j=1}^{J}\gamma^{j}=1$ needs to be added to programs (8) and (9) and their variations.

The dynamic Luenberger indicator as compared to the static Luenberger indicator has the advantage of accounting for dynamic linkages of production decisions over time and the presence of adjustment costs associated with investments in quasi-fixed factors of production. In contrast to other productivity change measures such as the Malmquist index which are built on the Shephard distance function, the Luenberger indicator is based on the directional distance function which generalizes Shephard distance functions. Directional distance function is based on the translation representation of the technology and thus Luenberger indicator is specified in difference form, on the contrary to Shephard distance function that is based on the radial technology (i.e., relative to the origin) and resulting Malmquist indexes being specified as ratios. The Luenberger indicators offer the special case of being interpreted also as radial measures of technology, offering the flexibility in choosing the directional vector in which input vectors are scaled. In addition, the ratio-based measures are very frequently not well defined in the neighbourhood of origin (i.e., using zero observations) which the difference based measures of Luenberger indicators overcome [28]. [29] present an analysis of the exact relations and specific conditions under which different productivity change measures are equal. Similar to almost all well-known productivity change measures, our dynamic Luenberger indicator suffers from the problem of infeasibility of mixed period distance functions which might occur when an observation from one period is beyond the production possibility set of the subsequent time period.

### Assessing the Impact of Regulation

Regulation may involve measures taking several years to be realized fully, and they may affect firms’ productivity for several years afterwards. In our analysis, we account for this by using dummy variables that capture the impact in each year after the implementation of the regulation. A similar approach is applied in regulation impact studies found in [30], [11]. As we also include two control variables of firms’ size and age, the estimated reduced form equation has the following form:

$$\begin{array}{l} y_{it}=\alpha_{i}+\beta_{0}\cdot R_{0}+\beta_{1}\cdot R_{1}+\beta_{2}\cdot R_{2}+\beta_{3}\cdot R_{3}+\beta_{4}\cdot R_{4}+\beta_{5}\cdot R_{5}+\beta_{6}\cdot R_{6}+\beta_{7}\cdot R_{7}+\\ \;\gamma_{1}\cdot SIZE_{it}+\gamma_{2}\cdot AGE_{it}+\varepsilon_{it} \end{array}$$ (10)

where *y*_*it*_ indicates the dynamic productivity growth (or its components) for a firm *i* in year *t*, *α*_*i*_ is a firm-specific constant, *R*_*0*—_ *R*_*7*_ are dummy variables indicating the number of years passed since the regulation was implemented; *SIZE*_*it*_ and *AGE*_*it*_ represent control factors of size and age for firm *i* in year *t*; *β*_0_ – *β*_7_ and *γ*_1_ − *γ*_2_ are coefficients to be estimated; and *ε*_*it*_ is an error term.

The equation is estimated using the OLS bootstrap regression with heteroskedasticity and autocorrelation robust standard errors. The use of robust standard errors accounts for possible problems with heteroskedasticity or clustered errors. The bootstrap approach is a method of estimating the distribution of the estimator through resampling. In the context of DEA efficiency scores, it is used to address the well-known problem of serial correlation among DEA scores [31]. Since efficiency scores are truncated, a truncated bootstrap regression approach is needed there. However, productivity indicators are not truncated, so in our context the OLS bootstrap regression is an appropriate approach. The current literature has already developed the bootstrap approach for directional distance function [32]. However, a bootstrap approach for dynamic directional distance function which forms the basis of dynamic Luenberger productivity indicators is non-existent in the literature. No bootstrap approach is available for static as well as dynamic Luenberger indicators, which could account for possible time dependence structure of the data as productivity change is measured between two time periods. Consequently, we do not apply bootstrap in the first stage when estimating the dynamic Luenberger indicator.

The robust Hausmann-Wooldridge test [33], [34] is used to discriminate between the random versus fixed effects models in the OLS bootstrap regression. The standard Hausman test [35] cannot be used because we apply heteroskedasticity and autocorrelation robust standard errors and bootstrap which violate the test’s requirements [36]. Eq (10) is estimated separately for dynamic productivity growth and each of its components as well as for meat processing, dairy processing and oils and fats firms.

### Data

Firm-level data are obtained from the SABI database, managed by Bureau van Dijk, which contains the financial accounts of Spanish firms classified according to the European industry classification system NACE. The study sample represents three activities of firms: meat processing (NACE Rev. 2 code 10.1), dairy processing (NACE Rev. 2 code 10.5) and oils and fats (NACE Rev. 2 code 10.4). Upon filtering out firms with missing observations and outliers, we are left with an unbalanced panel of 17,364 observations of meat processing firms, 4,141 observations of dairy processing firms and 3,250 observations of oils and fats firms for 1996–2011 period. Outliers were determined using ratios of output to input. An observation was defined as an outlier if the ratio of output over any of the three inputs was outside the interval of the median plus and minus two standard deviations.

The first step of our empirical strategy involves estimating the dynamic Luenberger productivity indicator and its components separately for meat processing, dairy processing and oils and fats firms. We consider one output, two variable inputs and one quasi-fixed input. Output is defined as total sales plus the change in the value of the stock and is deflated using the industrial price index for output in the meat processing industry, dairy processing industry and oils and fats, respectively. The two variable inputs are material and labour costs, which are taken directly from the SABI database and are deflated using the industrial price index for consumer non-durables and labour cost index in manufacturing, respectively. Fixed assets are considered a quasi-fixed input, measured as the beginning value of fixed assets from the balance sheet (i.e., the end value of the previous year) and are deflated using the industrial price index for capital goods. The Spanish Statistical Office is the source of all price indices used to deflate output and inputs. Gross investments in fixed assets in year *t* are computed as the beginning value of fixed assets in year *t+1* minus the beginning value of fixed assets in year *t* plus the beginning value of depreciation in year *t+1*. Table 1 reports the descriptive statistics of the input-output data used in this study, for the whole period 1996/1997-2010/2011 and indicates that the dairy processing firms and the oils and fats firms have an annual output that is, on average, more than twice the turnover of the meat processing firms. The standard deviations relative to their respective means are relatively high indicating that the firms in our sample differ considerably in size. Also, firms in the oils and fats industry have relatively low labour costs.

**Table 1:** Descriptive Statistics of the Data of the Spanish Meat Processing, Dairy Processing and Oils and Fats Industries, 1996–2011 (1000 Euro of 1995).

Variable	Meat processing industry	Dairy processing industry	Oils and fats industry
**Fixed assets**	1972.842 (14727.150)	4965.781 (23071.320)	4625.102 (40507.550)
**Labour cost**	603.352 (2997.394)	1286.860 (6280.649)	507.044 (2371.052)
**Material cost**	4897.449 (21934.990)	9022.490 (37032.050)	10553.360 (57681.250)
**Investments**	352.716 (4229.906)	683.734 (4127.369)	783.314 (11912.980)
**Output**	6655.046 (30287.400)	14933.120 (69203.200)	13173.090 (69233.320)

Note: Standard deviations are in parentheses.

The descriptive statistics of the variables used to analyse the relation between regulation and dynamic productivity growth and its components are presented in Table 2.

**Table 2:** Descriptive Statistics of Data Used in the Regression, 1996–2011.

Variable	Description	Meat processing industry	Dairy processing industry	Oils and fats industry
**Regulation age**	Dummies representing time since the beginning of regulation (since 2002)			
** R**_**0**_	Dummy = 1 for year 2002	0.076 (0.264)	0.073 (0.260)	0.072 (0.259)
** R**_**1**_	Dummy = 1 for year 2003	0.080 (0.272)	0.081 (0.274)	0.082 (0.274)
** R**_**2**_	Dummy = 1 for year 2004	0.083 (0.276)	0.083 (0.277)	0.085 (0.279)
** R**_**3**_	Dummy = 1 for year 2005	0.085 (0.279)	0.086 (0.280)	0.087 (0.282)
** R**_**4**_	Dummy = 1 for year 2006	0.087 (0.282)	0.082 (0.275)	0.090 (0.286)
** R**_**5**_	Dummy = 1 for year 2007	0.080 (0.271)	0.083 (0.276)	0.079 (0.269)
** R**_**6**_	Dummy = 1 for year 2008	0.077 (0.267)	0.089 (0.284)	0.077 (0.267)
** R**_**7**_	Dummy = 1 for years 2009–2011	0.160 (0.366)	0.192 (0.394)	0.182 (0.386)
**Size**	Dummies representing the firm’s size based on the number of employees and operating revenues			
** Micro**	Dummy = 1 for micro firms	0.401 (0.490)	0.518 (0.500)	0.463 (0.499)
** Small**	Dummy = 1 for small firms	0.449 (0.497)	0.317 (0.465)	0.389 (0.488)
** Medium**	Dummy = 1 for medium firms	0.119 (0.323)	0.102 (0.303)	0.109 (0.312)
** Large**	Dummy = 1 for large firms	0.031 (0.174)	0.064 (0.244)	0.039 (0.193)
**Age**	Number of years since the firm’s establishment	16.025 (9.535)	15.531 (11.276)	18.818 (15.089)

Note: Standard deviations are in parentheses.

In the absence of data on firm’s investment related with the implementation of the General Food Law, the impact of this regulation is captured through a set of variables *(R*_*0*_ *-R*_*7*_ *)* reflecting regulation age, measuring the number of years elapsed since the occurrence of regulation; i.e., since the 2002. The regulation age dummies range from 0 to 7-or-greater years old, where regulation age equal to 0 (dummy *R*_*0*_) takes the value 1 for the year 2002, while the dummy *R*_*7*_ takes the value 1 if the regulation took place 7 or more years ago. Regulation age equal to 7-or-greater years old serves as the reference category. The data in Table 2 indicate that the largest group of observations is captured by the 7-or-greater regulation age category.

*Age* and *Size* are two control factors used in the regression. *Size* is approximated by a set of dummy variables indicating four size categories of firms: micro, small, medium and large firms that are distinguished based on the EU definition of size. The EU size definition is based on the enterprises’ annual turnover and number of employees. In the regression, the category of large firms is taken as a reference. The data in Table 2 indicates that the majority of firms in meat processing, dairy processing and oils and fats firms represent the categories of micro and small firms. *Age* is measured as the number of years since firms’ establishment to the date of observation. The data in Table 2 indicate that, on average, firms in the oils and fats industry are the oldest, with the average firms’ age being nearly 19 years.

## Results and Discussion

The dynamic Luenberger productivity indicator and its decomposition are determined for each firm for a pair of consecutive years. The value of directional vector used in this article is (*g*_*x*_,*g*_*I*_) = (*x*,*δK*), i.e. *g*_*x*_ is the actual quantity of variable inputs and *g*_*I*_ is the depreciated quantity of capital (20% of capital stock).

As indicated before, the mixed period directional distance functions used to compute dynamic Luenberger indicator may yield infeasibilities. The most common method for the treatment of infeasibilities in the context of static Luenberger, which can be adapted to the dynamic context, is to exclude such observations in the computation of averages. We follow this strategy in this article. The infeasibilities we encounter in our computations account for 2% of meat processing industry observations, 5.6% for dairy processing industry observations and 10% for oils and fats industry observations.

### Dynamic Productivity Growth and its Decomposition

Table 3 presents the quartile specific and overall means of the dynamic Luenberger productivity indicator (*ΔL*) and its components of dynamic technical change (*ΔT*), dynamic technical inefficiency change (*ΔPEI*) and dynamic scale inefficiency change (*ΔSEI*) across industries. After computing Luenberger productivity growth, the firms are ranked according to magnitudes of their productivity growth and then they are grouped by quartiles ranging from the lowest (I) to the highest (IV). Then for each quartile the mean productivity growth is calculated. Note that the quartiles’ means for dynamic technical change, technical inefficiency change and scale inefficiency change are computed for Luenberger values of quartiles. The statistical test proposed by [37] is applied to assess the differences in Luenberger indicators and their components, which is based on the nonparametric test of the equality of two densities developed by [38].

**Table 3:** Dynamic Luenberger Productivity Change and its Components by Industry and Quartile Group (Mean Values Reported).

	Quartile group	Meat processing industry	Dairy processing industry	Oils and fats industry
**ΔL**	Lowest (I)	-0.089	-0.110	-0.182
	Lower middle (II)	-0.014	-0.017	-0.033
	Upper middle (III)	0.011	0.021	0.045
	Highest (IV)	0.080	0.106	0.199
	All	-0.003^a^	0.000^b^	0.007^c^
**ΔT**	Lowest (I)	-0.042	-0.014	-0.021
	Lower middle (II)	-0.040	-0.009	-0.016
	Upper middle (III)	-0.030	-0.012	-0.010
	Highest (IV)	-0.029	-0.010	-0.010
	All	-0.036^a^	-0.011^b^	-0.014^c^
**ΔPEI**	Lowest (I)	-0.061	-0.086	-0.156
	Lower middle (II)	0.010	-0.015	-0.009
	Upper middle (III)	0.033	0.025	0.043
	Highest (IV)	0.108	0.112	0.184
	All	0.022^a^	0.009^b^	0.015^c^
**ΔSEI**	Lowest (I)	0.014	-0.010	-0.005
	Lower middle (II)	0.016	0.006	-0.008
	Upper middle (III)	0.008	0.008	0.011
	Highest (IV)	0.001	0.004	0.026
	All	0.010^a^	0.002^b^	0.006^c^

Note: ^a,b,c^ denote significant differences between sectors at the critical 5% level.

The overall average dynamic Luenberger is very close to zero, from -0.3% per year for the meat processing industry to 0% for the dairy processing industry and 0.7% for the oils and fats industry. However, classifying firms based on their dynamic productivity change quartiles reveals considerable variation in dynamic productivity growth. For example, the meat processing firms in the lowest quartile have an average growth of -8.9%, while the highest quartile firms exhibit 8.0% dynamic productivity growth. The difference between the lowest and the highest quartiles show that dynamic Luenberger productivity growth is more dispersed across firms in the oils and fats industry than in the other two industries. The average dynamic Luenberger productivity indicators for the lowest and highest quartiles are approximately the same for the meat and dairy products sector, while the oils and fats sector presents dynamic productivity indictors for these same quartiles being approximately double those for meat and dairy products. The overall mean dynamic Luenberger productivity growth is significantly different between the sectors at the critical 5% level as shown by Li test results.

On average, the analysis of the components of dynamic productivity finds that dynamic technical change has a negative contribution to productivity growth, where dynamic technical inefficiency change and dynamic scale inefficiency change offer positive contributions. Technical regress is particularly high in the meat processing sector (-3.6% per year) and lower in the dairy processing (-1.1%) and oils and fats sector (-1.4%). Technical regress is also reported in other studies on the food processing industry. For the period 1996–2006, [13] found technical regress for the French cheese and poultry industry that can be attributed to the introduction of EU food regulation. Also, [39] found a negative technical change for Indian food industry over the period 1988–2005. Overall dynamic technical inefficiency change is positive suggesting that firms in each of the three sectors, on average use the existing production technology potential more efficiently over time. Overall average dynamic scale inefficiency change is positive, which suggests that firms have succeeded, on average, in moving the scale of the firm towards constant returns to scale.

A closer look at the distribution of the dynamic productivity growth components reveals a more subtle story. All quartiles of dynamic Luenberger productivity change across all sectors, on average, have a negative dynamic technical change contribution, which is the highest for the lowest quartile. The lowest quartile for the meat processing industry and the bottom two quartiles for both the dairy and oils and fats industries of dynamic Luenberger productivity change, on average, have a negative dynamic technical inefficiency change. Moreover, dynamic technical inefficiency change is widely dispersed across firms with dairy and oils and fats sectors presenting strong positive growth for the upper 50% of the distribution, and meat processing industry for the upper 75% of distribution. In particular, the oils and fats industry presents the most extreme increases and decreases of dynamic technical inefficiency over time. All quartiles of dynamic Luenberger productivity change have a positive dynamic scale inefficiency change for meat processing industry, all quartiles except of the lowest for dairy processing industry, and upper middle and the highest quartiles for oils and fats industry. Also, scale effect changes present the tightest distribution of all the dynamic Luenberger productivity components. Across all three industries, the positive dynamic productivity growth arises from the upper 50% of the distribution of dynamic technical inefficiency change and dynamic scale inefficiency change. Overall, the best productivity performers present technical regress, a strong positive technical inefficiency change, and a positive scale inefficiency change.

### Identifying the Impact of Regulatory Regime

Table 4 presents the results of regression for the impact of the introduction of the General Food Law in 2002 (Regulation (EC) No 178/2002) for dynamic Luenberger productivity indicator and its components for meat processing, dairy processing and oils and fats industry. The results reported here are those of the fixed effects model for all cases supported by the robust Hausman-Wooldridge test results.

**Table 4:** Results of the OLS Bootstrap Regression of Regulation Age and Control Variables on Dynamic Luenberger Productivity Growth and its Components.

	Meat processing industry				Dairy processing industry				Oils and fats industry			
	ΔL	ΔT	ΔPEI	ΔSEI	ΔL	ΔT	ΔPEI	ΔSEI	ΔL	ΔT	ΔPEI	ΔSEI
**Regulation age**												
**0 years old**	-2.6E-05	-0.068***	0.086***	-0.019***	0.002	0.028***	-0.031***	0.005	-0.059***	-0.112***	0.056***	-0.003
**1 year old**	0.003	0.038***	-0.016***	-0.020***	0.009*	0.008**	-0.028***	0.029***	-0.002	0.084***	-0.027**	-0.059***
**2 years old**	0.002	0.023***	0.007**	-0.028***	-0.005	-0.034***	0.009	0.021***	-0.061***	0.012*	-0.059***	-0.014**
**3 years old**	-0.002	0.011***	-0.018***	0.006***	-0.006	0.074***	-0.059***	-0.021***	-0.046***	-0.015**	-0.004	-0.028***
**4 years old**	0.004**	-0.054***	0.030***	0.028***	-0.020***	-0.041***	0.004	0.017***	0.094***	-0.114***	0.110***	0.097***
**5 years old**	-0.005**	-0.096***	0.077***	0.014***	-0.046***	-0.001	-0.003	-0.042***	-0.018	-0.035***	0.023**	-0.006
**6 years old**	0.001	0.024***	0.036***	-0.059***	0.035***	0.106***	-0.063***	-0.008**	0.098***	0.175***	-0.067***	-0.010*
**Size**												
** Micro**	0.025***	0.031**	0.059***	-0.065***	0.025	-0.008	0.028	0.006	-0.030	-0.081***	0.029	0.022
** Small**	0.020**	0.032***	0.047***	-0.059***	0.026*	-0.013	0.027	0.011	-0.027	-0.061**	0.012	0.022
**Medium**	0.011*	0.027**	0.030**	-0.046***	0.019	-0.008	0.017	0.010	-0.030	-0.029	-0.015	0.014
**Age**	1.0E-04	0.001***	-0.001***	-3.4E-04*	-6.9E-06	-0.005***	0.004***	0.001***	-0.005***	-0.010***	0.007***	-0.001**

Notes: ***, **, * denote significant at 1%, 5% and 10%, respectively. ΔL = dynamic productivity change; ΔT = dynamic technical change; ΔPEI = dynamic technical inefficiency change; ΔSEI = dynamic scale inefficiency change.

Dynamic productivity growth in the meat processing industry and dairy processing industry is only slightly affected by the introduction of the 2002 food regulation of the General Food Law: only two and four of the regulation age dummies are significant in meat and dairy, respectively. In the meat processing industry dynamic productivity growth is positively affected by the regulation in the fourth year and this is followed by a small negative effect in the year thereafter. Therefore, the effects are observed in the longer run. Similarly, for dairy processing firms the dynamic productivity growth first increases and then drops; however, it increases again in the final year. In the oils and fats industry regulation has a more pronounced impact on dynamic productivity growth. The coefficients of regulation ages 0, 2 and 3 years are significant and negative, while regulation ages that are 4 and 6 years old are significant and positive. Therefore, the 2002 General Food Law first reduces dynamic productivity growth and then increases dynamic productivity growth. These results for oils and fats industry are in line with the findings of [11]. It should be noted though that previous studies were conducted in a static context, so our results are not directly comparable.

Table 4 shows mixed evidence for each of the industries regarding the impact of regulation on technical change. In the meat processing and oils and fats industry the sign of the impact of regulation oscillates with the pattern observed as follows: dynamic technical change first decreases, then increases, then decreases again to increase in the final year. This result suggests that the regulation initially had a negative impact on dynamic technical change in these industries. Technical regress may result from the food safety regulation by increasing production costs, such as costs for additional hygiene measures and costs for implementing tracing systems, leading to organizational disruptions in implementing technologies. Such measures do not directly increase output, but merely increase production costs. The reverse pattern is observed for the dairy processing sector: dynamic technical change first increases, then trails off, then increases again, trails off again to finally increase in the 6^th^ year. Therefore, this result can imply that the increased stringency of food regulation initially improves dynamic technical change; i.e., spurs increased innovative activity by firms. Similar findings are reported in the study of [30] suggesting that environmental regulation enhances innovation.

Dynamic technical inefficiency change, in general, presents the reversed patterns compared to dynamic technical change in each of the industries. For meat processing and oils and fats firms, the contemporaneous impact of regulation (0 years old regulation) is positive, then dynamic technical inefficiency change decreases, then increases again, trails off again to finally increase in the 6^th^ year following the introduction of regulation (the final increase is not observed for oils and fats industry). The initial increase in dynamic technical inefficiency change may imply that the introduction of the food safety regulation may have induced meat processing and oils and fats firms to use the existing production potential more efficiently, as they were facing increasing production costs related to food safety measures. Hence, firms in these two industries have reacted to the regulation in a similar way exhibiting initially technical regress and firms dealing with economic stress from regulation to focus on improving technical efficiency, on average. For the dairy processing firms, the coefficient signs of regulation age dummies vary. However, only the negative coefficients are significant at the 1% critical level, suggesting an overall negative impact. Therefore, dynamic technical inefficiency change decreased in the initial years following the introduction of regulation and also in the longer run. In the actual year of implementation of the food regulation, the dairy processing industry is characterised by technological advances (increase in dynamic technical change contribution) along with a growth of the gap between efficient and inefficient firms (decrease in dynamic inefficiency change contribution). This may suggest that the initial decrease in dynamic technical inefficiency change is due to the failure of dairy firms to catch up with the technological improvements made by some of their competitors.

Table 4 shows mixed results between industries with regard to the impact of regulation on dynamic scale inefficiency change. The initial impact of regulation is negative for both meat processing and oils and fats firms. This result suggests that right after the introduction of the regulation firms had more difficulties in finding the optimal scale of operation. For oils and fats firms, this negative effect is maintained with an exception of one year. This indicates that the scale of operation has become less beneficial for the oils and fats industry even many years after the regulation was introduced. For meat processing firms, the decrease in dynamic scale inefficiency change recovers 3, 4 and 5 years post-food regulation introduction. On the other hand, dynamic scale inefficiency change in the dairy processing industry is impacted positively by regulation at first, suggesting that firms succeeded in moving to a firm scale consistent with constant returns to scale. However, in the 3^rd^, 5^th^ and 6^th^ year the impact of dynamic scale inefficiency change becomes negative in this industry.

As for the effects of control variables, the results in Table 4 show that in the dairy processing industry, size is generally not important for dynamic productivity growth nor for any of its components. An exception is a positive impact on dynamic productivity growth for small diary processing firms. However, size does matter in the meat processing industry and the results suggest that dynamic productivity growth, technical change and technical inefficiency change decrease with size. The opposite impact is observed for dynamic scale inefficiency change which is, *ceteris paribus*, higher on large firms. In the oils and fats industry, size proves to be important for dynamic technical change. In contrast to the meat processing firms, larger oils and fats firms exhibit a higher dynamic technical change, which may reflect that firms investing in a new technology grow and survive over the long run. The results for *Age* suggest that older meat processing firms, *ceteris paribus*, have higher dynamic technical change, and lower dynamic technical inefficiency change and dynamic scale inefficiency change. This result suggests that technical change is positively affected by the learning effect through age and experience. In the other two industries, dynamic technical change and technical inefficiency changes are negatively and positively affected by firm age, respectively. Hence, the learning effect has opposite impacts in the meat processing industry *vis-a-vis* the oils and fats, and dairy processing industries. However, in the oils and fats industry, scale inefficiency change decreases with age which is in line with the findings for meat processing industry.

## Conclusions

This article estimates dynamic Luenberger productivity growth of Spanish food processing firms over the period 1996–2011 and decomposes this growth into the contributions of dynamic technical inefficiency change, dynamic scale inefficiency change and dynamic technical change using Data Envelopment Analysis. A second stage econometric analysis applying the OLS regression with bootstrap is used to identify the impact of the introduction of the General Food Law in 2002 (Regulation (EC) No 178/2002) on dynamic productivity growth and its components.

The results show that while the dynamic Luenberger productivity growth was overall close to zero in the period 1996–2011, the components of productivity growth reveal the story. Dynamic technical change contributed negatively to productivity growth, on average, ranging between -1.1% for the dairy processing sector to -3.6% for the meat processing sector. Dynamic technical inefficiency change and scale inefficiency change made positive contributions, suggesting that firms used the production potential more efficiently and succeeded in moving closer to a scale of operation that is associated with constant returns to scale. However, we find that the distribution of the productivity growth components is quite broad. The highest quartile performers of dynamic technical inefficiency change and scale inefficiency change contribute positively. Dynamic technical change contributes negatively for all Luenberger quartile groups.

Firm level econometric estimates accounting for the long term impact of regulation confirm that in the meat processing industry the impact of General Food Law regulation on dynamic productivity growth is observed only in the longer run. However, the dairy processing and oils and fats firms present both short-term and long-term impacts of the 2002 food regulation. Overall, the results suggest that the impact of regulation on productivity growth could become less damaging and even positive. The results also suggest that the food regulation hampers dynamic technical change and dynamic scale inefficiency change in the short-term for meat processing and oils and fats firms. For dairy processing firms dynamic technical change and scale inefficiency change are impacted positively.

An interesting avenue for future research is to explore in more detail the effect of the food regulation on firms’ productivity. Our study uses time dummies to measure the impact of the regulation being introduced. However, time dummy variables may also pick up other coincident events. Hence, to analyse the effect of regulation more precisely, it would be useful to have data on the value of investments that were specifically undertaken by firms due to the regulation. Such data are difficult to acquire in databases available currently.

## Supporting Information

S1 Appendix Background on dynamic Luenberger indicator. (DOC)

S1 Data Protocol for downloading the raw data, cleaning dataset and creating variables. (DOC)
