# Supplementary material for: Effect of Food Regulation on the Spanish Food Processing Industry: A Dynamic Productivity Analysis
Source: PLoS One. 2015 Jun 9;10(6):e0128217. doi: 10.1371/journal.pone.0128217 (PMC4461303; doi:10.1371/journal.pone.0128217)
Supplement: S1 Appendix — (DOC) [file pone.0128217.s001.doc]

**Appendix**

At present, this appendix is included for the reviewers’ benefit to present the relevant background for the dynamic Luenberger productivity growth indicator decomposition into dynamic technical change and dynamic technical inefficiency change. The details of this decomposition presented here are found in [14].

The Luenberger indicator of productivity growth for the dynamic setting is defined for constant returns to scale in equation (2), with equation (3) presenting the contributions of dynamic technical inefficiency change (Δ*TEI*) and dynamic technical change (Δ*T*).

The decomposition of dynamic productivity growth is obtained from equation (2) by adding and subtracting the term , to yield:

or, rewritten as:

(A.1)

Dynamic technical inefficiency change is defined in equation (4) as the difference between the value of the dynamic directional distance function at time *t* and time *t+*1; i.e., the first term on the right hand side of (A.1). Dynamic technical inefficiency change is the difference between the distance functions evaluated using quantities and technologies in period *t* and period *t+1.* It measures the changes in the position of a firm relative to the dynamic production technology.

Dynamic technical change in equation (5) is the average distance between the two technologies at time *t* and time *t+*1*,* evaluated using quantities at time *t* [$\vec{D}_{t+1}^{i}(\mathbf{y}_{t},\mathbf{k}_{t},\mathbf{x}_{t},\mathbf{I}_{t};\mathbf{g}_{x},\mathbf{g}_{I})-$ $\vec{D}_{t}^{i}(\mathbf{y}_{t},\mathbf{k}_{t},\mathbf{x}_{t},\mathbf{I}_{t};\mathbf{g}_{x},\mathbf{g}_{I})$] and time *t+*1 [$\vec{D}_{t+1}^{i}(\mathbf{y}_{t+1},\mathbf{k}_{t+1},\mathbf{x}_{t+1},\mathbf{I}_{t+1};\mathbf{g}_{x},\mathbf{g}_{I})-$ $\vec{D}_{t+1}^{i}(\mathbf{y}_{t+1},\mathbf{k}_{t+1},\mathbf{x}_{t+1},\mathbf{I}_{t+1};\mathbf{g}_{x},\mathbf{g}_{I})$]. This is the second term on the right-hand side of (A.1). It represents the shift of dynamic production technology defined by the reduction of variable inputs and expansion of investments between two time period.
