# Supplementary material for: Effect of Food Regulation on the Spanish Food Processing Industry: A Dynamic Productivity Analysis
Source: PLoS One. 2015 Jun 9;10(6):e0128217. doi: 10.1371/journal.pone.0128217 (PMC4461303; doi:10.1371/journal.pone.0128217)
Supplement: S1 Data — (DOC) [file pone.0128217.s002.doc]

**Data protocol**

1. Downloading the raw data from the SABI database

- Search by:

Location – Country/Region in country – Spain

Industry – Industry classifications – Nace Rev.2. – 101 (Meat), 104 (Oils and fats), 105 (Dairy)

- Prepare the list format containing following variables: Company name, Date of establishment (Age), Fixed assets, Number of employees, Stocks, Operating revenues, Sales, Material costs, Cost of employees, Depreciation.
- Export data for searched firms according to the list format.

1. Cleaning dataset and creating variables using SAS software (using as an example the procedures for meat processing industry)
2. Deflation of variables, and computation of gross investments and production

IMPORT meat.xls */meat is an Excel file containing information on firms’ variables/*

IMPORT priceindexmeat.xls */priceindexmeat is an Excel file containing information on price index for variables/*

data meatpriceindex;

merge meat priceindexmeat;

by h;

run;

data meatdeflated (keep = QFa1995-QFa2011 Fa1995-Fa2011 Nemp1995-Nemp2011 QStocks1995-QStocks2011 Stocks1995-Stocks2011 Or1995-Or2011 QSales1995-QSales2011 Sales1995-Sales2011 QMcost1995-QMcost2011 Mcost1995-Mcost2011 QCostemp1995-QCostemp2011 Costemp1995-Costemp2011 QDepr1995-QDepr2011 Depr1995-Depr2011 Company Age Ginv1996-Ginv2011 Q2Ginv1996-Q2Ginv2011 Prod1996-Prod2011);

set meatpriceindex;

QFa1995 = Fa1995/Pc1995 *100;

QFa1996 = Fa1996/Pc1996 *100;

QFa1997 = Fa1997/Pc1997 *100;

QFa1998 = Fa1998/Pc1998 *100;

QFa1999 = Fa1999/Pc1999 *100;

QFa2000 = Fa2000/Pc2000 *100;

QFa2001 = Fa2001/Pc2001 *100;

QFa2002 = Fa2002/Pc2002 *100;

QFa2003 = Fa2003/Pc2003 *100;

QFa2004 = Fa2004/Pc2004 *100;

QFa2005 = Fa2005/Pc2005 *100;

QFa2006 = Fa2006/Pc2006 *100;

QFa2007 = Fa2007/Pc2007 *100;

QFa2008 = Fa2008/Pc2008 *100;

QFa2009 = Fa2009/Pc2009 *100;

QFa2010 = Fa2010/Pc2010 *100;

QFa2011 = Fa2011/Pc2011 *100;

QStocks1995 = Stocks1995/Ps1995 *100;

QStocks1996 = Stocks1996/Ps1996 *100;

QStocks1997 = Stocks1997/Ps1997 *100;

QStocks1998 = Stocks1998/Ps1998 *100;

QStocks1999 = Stocks1999/Ps1999 *100;

QStocks2000 = Stocks2000/Ps2000 *100;

QStocks2001 = Stocks2001/Ps2001 *100;

QStocks2002 = Stocks2002/Ps2002 *100;

QStocks2003 = Stocks2003/Ps2003 *100;

QStocks2004 = Stocks2004/Ps2004 *100;

QStocks2005 = Stocks2005/Ps2005 *100;

QStocks2006 = Stocks2006/Ps2006 *100;

QStocks2007 = Stocks2007/Ps2007 *100;

QStocks2008 = Stocks2008/Ps2008 *100;

QStocks2009 = Stocks2009/Ps2009 *100;

QStocks2010 = Stocks2010/Ps2010 *100;

QStocks2011 = Stocks2011/Ps2011 *100;

QSales1995 = Sales1995/Ps1995 *100;

QSales1996 = Sales1996/Ps1996 *100;

QSales1997 = Sales1997/Ps1997 *100;

QSales1998 = Sales1998/Ps1998 *100;

QSales1999 = Sales1999/Ps1999 *100;

QSales2000 = Sales2000/Ps2000 *100;

QSales2001 = Sales2001/Ps2001 *100;

QSales2002 = Sales2002/Ps2002 *100;

QSales2003 = Sales2003/Ps2003 *100;

QSales2004 = Sales2004/Ps2004 *100;

QSales2005 = Sales2005/Ps2005 *100;

QSales2006 = Sales2006/Ps2006 *100;

QSales2007 = Sales2007/Ps2007 *100;

QSales2008 = Sales2008/Ps2008 *100;

QSales2009 = Sales2009/Ps2009 *100;

QSales2010 = Sales2010/Ps2010 *100;

QSales2011 = Sales2011/Ps2011 *100;

QMcost1995 = Mcost1995/Pm1995 *100;

QMcost1996 = Mcost1996/Pm1996 *100;

QMcost1997 = Mcost1997/Pm1997 *100;

QMcost1998 = Mcost1998/Pm1998 *100;

QMcost1999 = Mcost1999/Pm1999 *100;

QMcost2000 = Mcost2000/Pm2000 *100;

QMcost2001 = Mcost2001/Pm2001 *100;

QMcost2002 = Mcost2002/Pm2002 *100;

QMcost2003 = Mcost2003/Pm2003 *100;

QMcost2004 = Mcost2004/Pm2004 *100;

QMcost2005 = Mcost2005/Pm2005 *100;

QMcost2006 = Mcost2006/Pm2006 *100;

QMcost2007 = Mcost2007/Pm2007 *100;

QMcost2008 = Mcost2008/Pm2008 *100;

QMcost2009 = Mcost2009/Pm2009 *100;

QMcost2010 = Mcost2010/Pm2010 *100;

QMcost2011 = Mcost2011/Pm2011 *100;

QCostemp1995 = Costemp1995/Pl1995 *100;

QCostemp1996 = Costemp1996/Pl1996 *100;

QCostemp1997 = Costemp1997/Pl1997 *100;

QCostemp1998 = Costemp1998/Pl1998 *100;

QCostemp1999 = Costemp1999/Pl1999 *100;

QCostemp2000 = Costemp2000/Pl2000 *100;

QCostemp2001 = Costemp2001/Pl2001 *100;

QCostemp2002 = Costemp2002/Pl2002 *100;

QCostemp2003 = Costemp2003/Pl2003 *100;

QCostemp2004 = Costemp2004/Pl2004 *100;

QCostemp2005 = Costemp2005/Pl2005 *100;

QCostemp2006 = Costemp2006/Pl2006 *100;

QCostemp2007 = Costemp2007/Pl2007 *100;

QCostemp2008 = Costemp2008/Pl2008 *100;

QCostemp2009 = Costemp2009/Pl2009 *100;

QCostemp2010 = Costemp2010/Pl2010 *100;

QCostemp2011 = Costemp2011/Pl2011 *100;

QDepr1995 = Depr1995/Pc1995 *100;

QDepr1996 = Depr1996/Pc1996 *100;

QDepr1997 = Depr1997/Pc1997 *100;

QDepr1998 = Depr1998/Pc1998 *100;

QDepr1999 = Depr1999/Pc1999 *100;

QDepr2000 = Depr2000/Pc2000 *100;

QDepr2001 = Depr2001/Pc2001 *100;

QDepr2002 = Depr2002/Pc2002 *100;

QDepr2003 = Depr2003/Pc2003 *100;

QDepr2004 = Depr2004/Pc2004 *100;

QDepr2005 = Depr2005/Pc2005 *100;

QDepr2006 = Depr2006/Pc2006 *100;

QDepr2007 = Depr2007/Pc2007 *100;

QDepr2008 = Depr2008/Pc2008 *100;

QDepr2009 = Depr2009/Pc2009 *100;

QDepr2010 = Depr2010/Pc2010 *100;

QDepr2011 = Depr2011/Pc2011 *100;

if (Fa1996 gt 0 and Fa1995 gt 0 and Depr1996 gt 0) then do

Ginv1996=Fa1996-Fa1995+Depr1996;

end;

if (Fa1997 gt 0 and Fa1996 gt 0 and Depr1997 gt 0) then do

Ginv1997=Fa1997-Fa1996+Depr1997;

end;

if (Fa1998 gt 0 and Fa1997 gt 0 and Depr1998 gt 0) then do

Ginv1998=Fa1998-Fa1997+Depr1998;

end;

if (Fa1999 gt 0 and Fa1998 gt 0 and Depr1999 gt 0) then do

Ginv1999=Fa1999-Fa1998+Depr1999;

end;

if (Fa2000 gt 0 and Fa1999 gt 0 and Depr2000 gt 0) then do

Ginv2000=Fa2000-Fa1999+Depr2000;

end;

if (Fa2001 gt 0 and Fa2000 gt 0 and Depr2001 gt 0) then do

Ginv2001=Fa2001-Fa2000+Depr2001;

end;

if (Fa2002 gt 0 and Fa2001 gt 0 and Depr2002 gt 0) then do

Ginv2002=Fa2002-Fa2001+Depr2002;

end;

if (Fa2003 gt 0 and Fa2002 gt 0 and Depr2003 gt 0) then do

Ginv2003=Fa2003-Fa2002+Depr2003;

end;

if (Fa2004 gt 0 and Fa2003 gt 0 and Depr2004 gt 0) then do

Ginv2004=Fa2004-Fa2003+Depr2004;

end;

if (Fa2005 gt 0 and Fa2004 gt 0 and Depr2005 gt 0) then do

Ginv2005=Fa2005-Fa2004+Depr2005;

end;

if (Fa2006 gt 0 and Fa2005 gt 0 and Depr2006 gt 0) then do

Ginv2006=Fa2006-Fa2005+Depr2006;

end;

if (Fa2007 gt 0 and Fa2006 gt 0 and Depr2007 gt 0) then do

Ginv2007=Fa2007-Fa2006+Depr2007;

end;

if (Fa2008 gt 0 and Fa2007 gt 0 and Depr2008 gt 0) then do

Ginv2008=Fa2008-Fa2007+Depr2008;

end;

if (Fa2009 gt 0 and Fa2008 gt 0 and Depr2009 gt 0) then do

Ginv2009=Fa2009-Fa2008+Depr2009;

end;

if (Fa2010 gt 0 and Fa2009 gt 0 and Depr2010 gt 0) then do

Ginv2010=Fa2010-Fa2009+Depr2010;

end;

if (Fa2011 gt 0 and Fa2010 gt 0 and Depr2011 gt 0) then do

Ginv2011=Fa2011-Fa2010+Depr2011;

end;

Q2Ginv1996 = Ginv1996/Pc1996 *100;

Q2Ginv1997 = Ginv1997/Pc1997 *100;

Q2Ginv1998 = Ginv1998/Pc1998 *100;

Q2Ginv1999 = Ginv1999/Pc1999 *100;

Q2Ginv2000 = Ginv2000/Pc2000 *100;

Q2Ginv2001 = Ginv2001/Pc2001 *100;

Q2Ginv2002 = Ginv2002/Pc2002 *100;

Q2Ginv2003 = Ginv2003/Pc2003 *100;

Q2Ginv2004 = Ginv2004/Pc2004 *100;

Q2Ginv2005 = Ginv2005/Pc2005 *100;

Q2Ginv2006 = Ginv2006/Pc2006 *100;

Q2Ginv2007 = Ginv2007/Pc2007 *100;

Q2Ginv2008 = Ginv2008/Pc2008 *100;

Q2Ginv2009 = Ginv2009/Pc2009 *100;

Q2Ginv2010 = Ginv2010/Pc2010 *100;

Q2Ginv2011 = Ginv2011/Pc2011 *100;

Prod1996=QSales1996+QStocks1996-QStocks1995;

Prod1997=QSales1997+QStocks1997-QStocks1996;

Prod1998=QSales1998+QStocks1998-QStocks1997;

Prod1999=QSales1999+QStocks1999-QStocks1998;

Prod2000=QSales2000+QStocks2000-QStocks1999;

Prod2001=QSales2001+QStocks2001-QStocks2000;

Prod2002=QSales2002+QStocks2002-QStocks2001;

Prod2003=QSales2003+QStocks2003-QStocks2002;

Prod2004=QSales2004+QStocks2004-QStocks2003;

Prod2005=QSales2005+QStocks2005-QStocks2004;

Prod2006=QSales2006+QStocks2006-QStocks2005;

Prod2007=QSales2007+QStocks2007-QStocks2006;

Prod2008=QSales2008+QStocks2008-QStocks2007;

Prod2009=QSales2009+QStocks2009-QStocks2008;

Prod2010=QSales2010+QStocks2010-QStocks2009;

Prod2011=QSales2011+QStocks2011-QStocks2010;

run;

1. Preparing datasets for each year

*/1996/*

data meat1996 (keep = QFa1995 QMcost1996 QCostemp1996 QDepr1996 Company Size Age Q2Ginv1996 Prod1996);

set meatdeflated;

if Or1996 le 2000 and Nemp1996 lt 10 then Size=1;

if Or1996 gt 2000 and Nemp1996 lt 10 then Size=2;

if Or1996 le 2000 and Nemp1996 ge 10 then Size=2;

if (Or1996 le 10000 and Or1996 gt 2000) and (Nemp1996 lt 50 and Nemp1996 ge 10) then Size=2;

if (Or1996 gt 10000) and (Nemp1996 lt 50 and Nemp1996 ge 10) then Size=3;

if (Or1996 le 10000 and Or1996 gt 2000) and (Nemp1996 ge 50) then Size=3;

if (Or1996 le 50000 and Or1996 gt 10000) and (Nemp1996 lt 250 and Nemp1996 ge 50) then Size=3;

if (Or1996 gt 50000) and (Nemp1996 lt 250 and Nemp1996 ge 50) then Size=4;

if (Or1996 le 50000 and Or1996 gt 10000) and (Nemp1996 ge 250) then Size=4;

if Or1996 gt 50000 and Nemp1996 ge 250 then Size=4;

run;

data meatlimpio1996;

set meat1996;

if Prod1996 = . or Prod1996 le 0 then delete;

if Q2Ginv1996 = . then delete;

if QMcost1996 = . or QMcost1996 le 0 then delete;

if QCostemp1996 = . or QCostemp1996 le 0 then delete;

if QFa1995 = . or QFa1995 le 0 then delete;

if Size = . delete;

run;

data meatproductivity1996 (keep = QFa1995 QMcost1996 QCostemp1996 QDepr1996 Company Size Age Q2Ginv1996 Prod1996 PF1996 PCE1996 PMC1996 h);

set meatlimpio1996;

PF1996 = Prod1996 / QFa1995;

PCE1996 = Prod1996 / QCostemp1996;

PMC1996 = Prod1996 / QMcost1996;

h=1;

run;

proc means data=meatproductivity1996;

var PF1996 PCE1996 PMC1996 h;

output out=mmeat1996 median=MPF1996 MPCE1996 MPMC1996 h std=SPF1996 SPCE1996 SPMC1996 sh;

run;

data meatcleanout1996median2 (keep = QFa1995 QMcost1996 QCostemp1996 QDepr1996 Company Size Age Q2Ginv1996 Prod1996 PF1996 PCE1996 PMC1996);

merge mmeat1996 meatproductivity1996;

by h;

if PF1996 le MPF1996-2*SPF1996 then delete;

if PF1996 ge MPF1996 +2*SPF1996 then delete;

if PCE1996 le MPCE1996-2*SPCE1996 then delete;

if PCE1996 ge MPCE1996+2*SPCE1996 then delete;

if PMC1996 le MPMC1996-2*SPMC1996 then delete;

if PMC1996 ge MPMC1996+2*SPMC1996 then delete;

run;

data meat1996final;

set meatcleanout1996median2;

QFa=QFa1995;

QMcost=QMcost1996;

QCostemp=QCostemp1996;

QDepr=QDepr1996;

Q2Ginv=Q2Ginv1996;

Prod=Prod1996;

Year=1996;

drop QFa1995;

drop QMcost1996;

drop QCostemp1996;

drop QDepr1996;

drop Q2Ginv1996;

drop Prod1996;

run;

/1997/

data meat1997 (keep = QFa1996 QMcost1997 QCostemp1997 QDepr1997 Company Size Age Q2Ginv1997 Prod1997);

set meatdeflated;

if Or1997 le 2000 and Nemp1997 lt 10 then Size=1;

if Or1997 gt 2000 and Nemp1997 lt 10 then Size=2;

if Or1997 le 2000 and Nemp1997 ge 10 then Size=2;

if (Or1997 le 10000 and Or1997 gt 2000) and (Nemp1997 lt 50 and Nemp1997 ge 10) then Size=2;

if (Or1997 gt 10000) and (Nemp1997 lt 50 and Nemp1997 ge 10) then Size=3;

if (Or1997 le 10000 and Or1997 gt 2000) and (Nemp1997 ge 50) then Size=3;

if (Or1997 le 50000 and Or1997 gt 10000) and (Nemp1997 lt 250 and Nemp1997 ge 50) then Size=3;

if (Or1997 gt 50000) and (Nemp1997 lt 250 and Nemp1997 ge 50) then Size=4;

if (Or1997 le 50000 and Or1997 gt 10000) and (Nemp1997 ge 250) then Size=4;

if Or1997 gt 50000 and Nemp1997 ge 250 then Size=4;

run;

data meatlimpio1997;

set meat1997;

if Prod1997 = . or Prod1997 le 0 then delete;

if Q2Ginv1997 = . then delete;

if QMcost1997 = . or QMcost1997 le 0 then delete;

if QCostemp1997 = . or QCostemp1997 le 0 then delete;

if QFa1996 = . or QFa1996 le 0 then delete;

if Size = . delete;

run;

data meatproductivity1997 (keep = QFa1996 QMcost1997 QCostemp1997 QDepr1997 Company Size Age Q2Ginv1997 Prod1997 PF1997 PCE1997 PMC1997 h);

set meatlimpio1997;

PF1997 = Prod1997 / QFa1996;

PCE1997 = Prod1997 / QCostemp1997;

PMC1997 = Prod1997 / QMcost1997;

h=1;

run;

proc means data=meatproductivity1997;

var PF1997 PCE1997 PMC1997 h;

output out=mmeat1997 median=MPF1997 MPCE1997 MPMC1997 h std=SPF1997 SPCE1997 SPMC1997 sh;

run;

data meatcleanout1997median2 (keep = QFa1996 QMcost1997 QCostemp1997 QDepr1997 Company Size Age Q2Ginv1997 Prod1997 PF1997 PCE1997 PMC1997);

merge mmeat1997 meatproductivity1997;

by h;

if PF1997 le MPF1997-2*SPF1997 then delete;

if PF1997 ge MPF1997 +2*SPF1997 then delete;

if PCE1997 le MPCE1997-2*SPCE1997 then delete;

if PCE1997 ge MPCE1997+2*SPCE1997 then delete;

if PMC1997 le MPMC1997-2*SPMC1997 then delete;

if PMC1997 ge MPMC1997+2*SPMC1997 then delete;

run;

data meat1997final;

set meatcleanout1997median2;

QFa=QFa1996;

QMcost=QMcost1997;

QCostemp=QCostemp1997;

QDepr=QDepr1997;

Q2Ginv=Q2Ginv1997;

Prod=Prod1997;

Year=1997;

drop QFa1996;

drop QMcost1997;

drop QCostemp1997;

drop QDepr1997;

drop Q2Ginv1997;

drop Prod1997;

run;

*/The same as above should be done for all remaining years i.e.1998, 1999 etc./*

1. Creating datasets for computing Luenberger indicator

*/1996/1997/*

PROC SORT DATA =meat1996final;

BY DMU;

RUN;

PROC SORT DATA =meat1997final;

BY DMU;

RUN;

DATA FIRMS9697;

set meat1996final meat1997final;

BY DMU;

RUN;

PROC MEANS DATA =FIRMS9697 NOPRINT;

BY DMU;

VAR QFA;

OUTPUT OUT = MEAN9697 MEAN=MQFA2000;

RUN;

DATA MFIRMS9697;

MERGE FIRMS9697 MEAN9697;

BY DMU;

IF _FREQ_ =1 then delete;

run;

proc sort data =mfirms9697;

by year;

run;

data meat9697 (keep=P MC EC F I D);

set mfirms9697;

P = Prod / 1000;

MC = QMcost / 1000;

EC = QCostemp / 1000;

F = QFa / 1000;

I = Q2Ginv / 1000;

D = QDepr / 1000;

run;

data _null_;

set meat9697;

file 'meat9697.txt';

if _N_ ge 1 then put

(_N_) (10.0) (P) (15.3) (MC) (15.3) (EC) (15.3) (F) (15.3) (I) (15.3) (D) (15.3);

run;

*/The same as above should be done for all pairs of years i.e. 1997/1998, 1998/1999 etc./*

*/Finally all procedures described in (2) should be repeated for dairy processing, and oils and fats industries./*
